# Functional Microbial Responses to Alcohol Abstinence in Patients With Alcohol Use Disorder

**DOI:** 10.3389/fphys.2020.00370

**Published:** 2020-04-24

**Authors:** Bei Gao, Atoosa Emami, Rongrong Zhou, Sonja Lang, Yi Duan, Yanhan Wang, Lu Jiang, Rohit Loomba, David A. Brenner, Peter Stärkel, Bernd Schnabl

**Affiliations:** ^1^Department of Medicine, University of California, San Diego, San Diego, CA, United States; ^2^Department of Medicine, VA San Diego Healthcare System, San Diego, CA, United States; ^3^St. Luc University Hospital, Université Catholique de Louvain, Brussels, Belgium

**Keywords:** microbiome, metagenomics, steatosis, AUD, CAP

## Abstract

Excessive alcohol consumption is associated with hepatic steatosis and dysregulation of the gut microbiota in patients with alcohol use disorder (AUD). However, how gut microbiota responds when patients stop drinking has not been well studied. In this study, we use shotgun metagenomic sequencing to elucidate the alterations in the functional capacity of gut microbiota in patients with AUD when they stop drinking for 2-weeks. Sensitive microbial pathways to alcohol abstinence were identified in AUD patients. Further, we found the functional microbial responses to alcohol abstinence were different in AUD patients with different degree of hepatic steatosis. Our results provide insights into the link between functional alterations of the gut microbiota and steatosis associated with alcohol consumption.

## Introduction

Alcohol-related health problems are a major medical burden worldwide. In response to moderate to large amounts of alcohol consumption, steatosis develops in 90–95% of patients with alcohol-use disorder (EASL, 2012). Controlled attenuation parameter (CAP) is a non-invasive method to detect hepatic steatosis using transient elastography (Myers et al., 2012). CAP values range from 100 to 400 dB/m, where higher values indicate more severe steatosis (Mikolasevic et al., 2016). In addition to steatosis, chronic and excessive alcohol consumption contributes to gut barrier dysfunction, increased intestinal permeability, and dysregulation of the gut microbiota (Szabo, 2015). The gut metagenome, encoding 100-fold more unique genes than human genome, has a vast metabolic capacity. Metagenomic analysis of the gut microbiota reveals the functional capacity of the gut community and allows for further understanding of their potential metabolic functions (Qin et al., 2010).

The impact of alcohol on the composition of the gut microbiota has been reported previously (Qin et al., 2010; Mutlu et al., 2012; Bull-Otterson et al., 2013; Dubinkina et al., 2017; Wang et al., 2018). However, how the gut metagenome responds when alcohol use disorder (AUD) patients stop drinking has not been investigated. In this study, we use shotgun metagenomic sequencing to elucidate the alterations in the functional capacity of gut microbiota in AUD patients when they stop drinking for 2 weeks. Our aim is to identify sensitive microbial pathways to alcohol abstinence in AUD patients.

## Materials and Methods

### Patients

Patient cohorts have been described (Brandl et al., 2018; Duan et al., 2019; Gao et al., 2019; Lang et al., 2019). Eight (8) non-alcoholic controls and 30 patients with AUD were included in this study. AUD patients were recruited from an alcohol withdrawal unit where they followed a highly standardized and controlled 3-weeks detoxification and rehabilitation program consisting of 1 week inpatient detoxification (day 1–7) followed by 1 week outpatient care (day 8–14) and 1 week hospital readmission for consolidation (day 15–21). The diagnosis of AUD was based on the DSM IV criteria (Ball et al., 1997). Patients reported long-term (>1 year) alcohol consumption >60 g/day and were actively drinking until the day of admission for elective alcohol withdrawal. Complete physical examination, medication and medical history are taken at admission. Serum samples were collected, and patients underwent FibroScan^®^ (Echosens, Paris, France) with determination of liver stiffness and continued attenuation parameter (CAP) measurements on the day of admission. Stool samples were collected from the first bowel movement after each hospital admission. These samples and procedures were labeled “WEEK 1” for the initial hospitalization and “WEEK 3” after hospital readmission. We collected fecal samples from spontaneous bowel movements of patients, and timing of these evacuations varies depending on the patient. Because of this, the collection time was not precisely controlled, and diurnal variations in fecal microbiota may contribute to the variations in the metagenomics data, which was one limitation of this study. Non-alcoholic controls were social drinkers consuming less than 20 g of alcohol per day, which was one inclusion criteria. Non-alcoholic controls and AUD patients did not take antibiotics or immunosuppressive medication during the 2 months preceding enrollment. Other exclusion criteria were diabetes, inflammatory bowel disease, known liver disease of any other etiology, and clinically significant cardio-vascular, pulmonary or renal co-morbidities. Written informed consent was signed by each participant after the nature and possible consequences of the studies were explained. The study protocol was approved by the Institutional Review Board from each institution involved.

### Continued Attenuation Parameter (CAP) Measurements

Liver steatosis was assessed non-invasively by CAP (Nguyen-Khac et al., 2018). The measurements were performed with the Fibroscan^®^ device (Echosens, Paris, France) by an experienced examiner blinded to patient’s data. CAP was deemed valid if at least ten validated measurements were obtained with an interquartile range ≤40 dB/m. Final results were the median of all obtained valid measurements. The patients were divided into CAP High group (≥300 dB/m, severe steatosis, *n* = 13) and CAP Low group (<300 dB/m, *n* = 17). This cutoff was derived from recent studies performed in NAFLD patients (Buzzetti et al., 2015; Karlas et al., 2017; Caussy et al., 2018; Eddowes et al., 2019).

### Shotgun Metagenomic Analysis

DNA was extracted from fecal samples using FastDNA Spin Kit (MP-Biomedicals). Shotgun metagenomic sequencing was performed on Illumina HiSeq 4000 generating 150 bp paired-end reads. KneadData version 0.7.2 was used for the quality control of shotgun metagenomic raw sequencing reads. MetaPhlAn2 version 2.7.7 and HUMAnN2 version 0.11.1 was used for the profiling of composition of the microbial communities and microbial pathways, respectively, (Truong et al., 2015; Franzosa et al., 2018). Each of the HUMAnN2 abundance output was normalized into relative abundance.

### Short-Chain Fatty Acids Measurement

Short-chain fatty acids extraction and quantification was performed as described in our previous study (Nusbaum et al., 2018). Briefly, short-chain fatty acids were extracted from 10 mg feces with 700 μL of 5:1:1 water, hydrochloric acid and methyl tert-butyl ether. Samples were homogenized and centrifuged. Supernatant underwent dehydration and tert-butyldimethylsilylation with tert-Butyldimethylsilyl-N-methyltrifluoroacetamide (MTBSTFA, Sigma-Aldrich). Samples were shaken at 80°C for 30 min and injected into an Agilent 5977A GC-quadrupole mass spectrometer with selected ion monitoring mode. Agilent Mass Hunter Quantitative Analysis software (B.07.00) was used for data processing. Data were quantified against authentic standards.

### Statistical Analysis

Linear discriminant analysis effect size (LEfSe) was used for the biomarker discovery (Segata et al., 2011). For LEfSe analysis, all-against-all strategy was used. Alpha value for the factorial Kruskal–Wallis test among classes was set to 0.05. Threshold on the logarithmic linear discriminant analysis (LDA) score for discriminative features was set to 2.0. For the statistical analysis of butyrate concentration, wilcoxon rank-sum test was used. Spearman correlation was used to test the correlation between two variables.

## Results

### Patient Characteristics

Serum and fecal samples were collected from 8 non-alcoholic controls and 30 patients with AUD. The AUD patients were divided into CAP Low (*n* = 17) and CAP High (*n* = 13) groups as defined by a CAP cutoff of 300 dB/m (Buzzetti et al., 2015; Karlas et al., 2017; Caussy et al., 2018; Eddowes et al., 2019). The baseline characteristics of patients with AUD and control subjects are shown in Supplementary Table S1. Subjects were predominantly males, and there was no difference in gender and age when compared between controls and AUD patients. The BMI was not different between control and patients with AUD (CAP Low and High), but patients in CAP Low group had a higher BMI than CAP High group (*p*-value = 0.02). Even though, BMI is still within normal range. There was no difference between CAP Low and High groups for the comparison of laboratory parameters including albumin, aspartate aminotransferase (AST), alanine aminotransferase (ALT), gamma-glutamyl transferase (GGT), total bilirubin, platelet counts, creatinine, and international normalized ratio (INR).

### Sensitive Microbial Pathways to Alcohol

**FIGURE 1:**
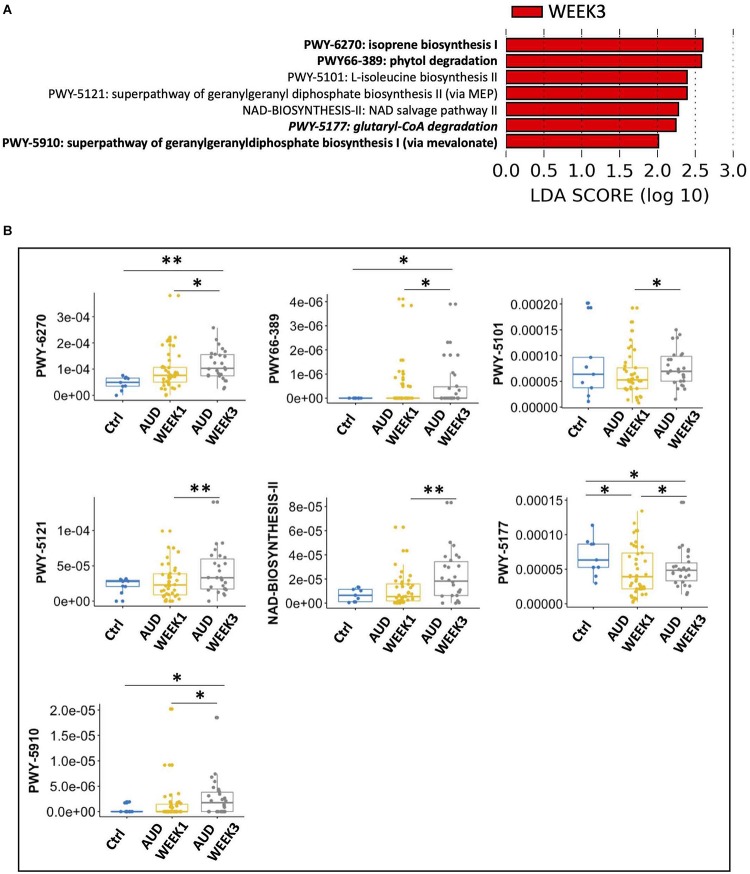
Seven sensitive microbial pathways to alcohol abstinence in alcohol use disorder patients. **(A)** Seven microbial pathways were enriched at WEEK 3 compared with WEEK 1. Italic: significantly altered pathway comparing control subjects and alcohol use disorder patients at WEEK 1. Bold: significantly altered pathways comparing control subjects and alcohol use disorder patients at WEEK 3. **(B)** Pairwise comparison of control subjects, alcohol use disorder patients at WEEK 1 and alcohol use disorder patients at WEEK 3. ^∗^: *p*-value < 0.05; ^∗∗^: *p*-value < 0.01.

To identify the sensitive microbial pathways, we compared the gut microbial pathway in AUD patients at WEEK 1 and WEEK 3. A total of seven microbial pathways were sensitive to alcohol abstinence with LDA score > 2.0, all of which were enriched at WEEK 3 (Figure 1A), including isoprene biosynthesis I, phytol degradation, L-isoleucine biosynthesis II, superpathway of geranylgeranyl diphosphate biosynthesis II (via MEP), NAD salvage pathway II, glutaryl-CoA degradation and superpathway of geranylgeranyldiphosphate biosynthesis I (via mevalonate).

Furthermore, to investigate whether these seven sensitive microbial pathways were also different between AUD patients and control subjects, we compared all detected microbial pathways between control subjects and AUD patients at WEEK 1 and WEEK 3, respectively. At WEEK 1, a total of 34 and 22 microbial pathways were enriched in AUD patients and control subjects, respectively (Supplementary Figure S1). Notably, glutaryl-CoA degradation was enriched in control subjects, which was one of the sensitive microbial pathways enriched in AUD patients at WEEK 3 (Figure 1B). Next, we compared microbial pathways between control subjects and AUD patients at WEEK 3 and found 46 and 17 microbial pathways were enriched in AUD patients and control subjects, respectively (Supplementary Figure S2). Four sensitive microbial pathways were found, among which three were enriched in AUD patients at WEEK 3, including isoprene biosynthesis I, phytol degradation, and superpathway of geranylgeranyldiphosphate biosynthesis I (via mevalonate). Meanwhile, glutaryl-CoA degradation was enriched in control subjects, which was also enriched in control subjects when comparing controls with AUD patients at WEEK 1 (Figure 1B). The remaining three microbial pathways were not significantly different between control subjects and AUD patients either at WEEK 1 or WEEK 3, including L-isoleucine biosynthesis II, superpathway of geranylgeranyl diphosphate biosynthesis II (via MEP) and NAD salvage pathway II (Figure 1B).

### Different Microbial Functional Responses in CAP High and Low Patients

**FIGURE 2:**
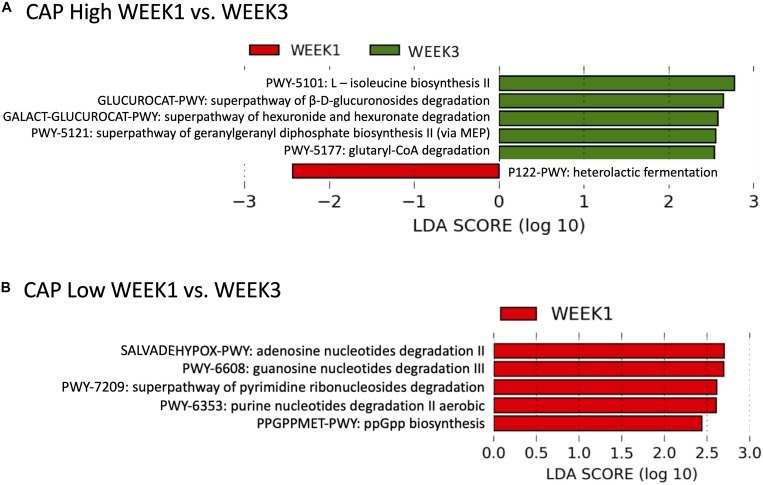
Functional microbial response to alcohol abstinence were different in CAP High and CAP Low patients. **(A)** In CAP High patients, five microbial pathways were enriched at WEEK 3 (Green) and one microbial pathways were enriched at WEEK 1 (Red). **(B)** In CAP Low patients, five microbial pathways were enriched at WEEK 1 (Red).

The functional microbial responses to alcohol abstinence were different in CAP High (≥300 dB/m) and CAP Low (<300 dB/m) patients. Five microbial pathways were significantly increased at WEEK 3 in CAP High patients with LDA score > 2.0, including L-isoleucine biosynthesis II, superpathway of β-D-glucuronosides degradation, superpathway of hexuronide and hexuronate degradation, superpathway of geranylgeranyl diphosphate biosynthesis II (via MEP), and glutaryl-CoA degradation (Figure 2A). In contrast, heterolactic fermentation was decreased at WEEK 3 in CAP High patients (LDA score > 2.0, Figure 2A). When comparing WEEK 1 and WEEK 3 microbial pathways in CAP Low patients, we found five microbial pathways were enriched at WEEK 1 (LDA score > 2.0, Figure 2B), including adenosine nucleotides degradation II, guanosine nucleotides degradation III, superpathway of pyrimidine ribonucleosides degradation, purine nucleotides degradation II aerobic, and ppGpp biosynthesis.

## Discussion

By comparing the WEEK 3 metagenomic sequencing data with WEEK 1 in AUD patients, we found seven sensitive pathways, among which four pathways were different when comparing control subjects with AUD patients at WEEK 1 or WEEK 3. Especially, the relative abundance of glutaryl-CoA degradation was lower in AUD patients at WEEK 1 when compared with controls and it was increased in AUD patients at WEEK 3 compared with AUD patients at WEEK 1. However, its relative abundance was still lower at WEEK 3 when comparing with controls (Figure 1B). During glutaryl-CoA degradation process, glutaryl-CoA is turned into acetyl-CoA through β-oxidation, which functions as an intermediate in many metabolic pathways. *F. prausnitzii* is a major contributor to glutaryl-CoA degradation (Supplementary Figure S3A). *F. prausnitzii* is one of the most abundant species in the human microbiome (Martín et al., 2017). In the present study, AUD patients showed a lower relative abundance of *F. prausnitzii* compared with control group (Supplementary Figure S3B), which is consistent with previous findings in alcohol-dependent subjects (Leclercq et al., 2014). A high-fat diet feeding mouse model shows that *F. prausnitzii* supplementation improves hepatic health and reduces adipose tissue inflammation (Munukka et al., 2017). *F. prausnitzii* is known as a butyrate producer. Therefore, we measured the level of short-chain fatty acids in patients with AUD and then performed spearman correlation between short-chain fatty acids and *F. prausnitzii*. We found increased *F. prausnitzii* was associated with elevated butyrate level in fecal samples (Supplementary Figure S3C). We further checked the fecal level of butyrate in non-alcoholic controls and AUD patients, however, no significant difference was found (Supplementary Figure S3D).

The microbial functional responses to alcohol abstinence were different in CAP High (≥300 dB/m) and CAP Low (<300 dB/m) patients. Among the six significantly different microbial pathways in CAP High patients, β-glucuronidase was involved in two processes, including superpathway of β-D-glucuronosides degradation and superpathway of hexuronide and hexuronate degradation. During the superpathway of β-D-glucuronosides degradation process, β-glucuronosides undergo hydrolysis by β-glucuronidase to yield D-glucuronate. Similarly to the superpathway of β-D-glucuronosides degradation, β-glucuronidase is also the first enzyme utilized in the degradation of hexuronide and hexuronate (Mandrand-Berthelot et al., 2004). Besides, L- isoleucine biosynthesis II was increased in the CAP High patients at WEEK 3 compared with WEEK 1. Branched chain amino acids including isoleucine have been found to alleviate steatosis by suppressing gene and protein expression level of fatty acid synthase (Honda et al., 2017). Rats deficient in isoleucine had higher levels of triglyceride in the liver compared with rats with normal levels (Lyman et al., 1964). While in CAP Low patients, the microbial processes involved in nucleotides and nucleosides degradation was lower at WEEK 3. Different microbial functional responses to alcohol abstinence in CAP High and Low patients suggested the complex interplay between alcohol, microbiota and the hepatic steatosis.

In summary, we identified sensitive microbial pathways to alcohol abstinence and found different microbial functional responses to alcohol abstinence in CAP High and Low patients. Our study provides insights into the link between functional alterations of the gut microbiota and steatosis induced by alcohol consumption. One limitation of our study is that the sample size is small and these findings need to be validated in a larger patient cohort.

## Data Availability Statement

The sequencing data can be found in the BioProject database (accession:PRJNA613834, https://www.ncbi.nlm.nih.gov/Traces/study/?acc=PRJNA613834).

## Ethics Statement

The studies involving human participants were reviewed and approved by The study protocol was approved by the Institutional Review Board from each institution involved. The patients/participants provided their written informed consent to participate in this study.

## Author Contributions

BG was responsible for data analysis and manuscript editing. AE was responsible for manuscript writing. RZ, SL, and LJ were responsible for the analysis of clinical data. YD and YW were responsible for sample preparation. RL and DB were responsible for manuscript editing. PS and BS were responsible for the study concept and design, manuscript editing, and study supervision.

## Conflict of Interest

BS has been consulting for Ferring Research Institute, Intercept Pharmaceuticals, HOST Therabiomics, and Patara Pharmaceuticals. BS’s institution UC San Diego has received grant support from BiomX, NGM Biopharmaceuticals, CymaBay Therapeutics, and Synlogic Operating Company. The remaining authors declare that the research was conducted in the absence of any commercial or financial relationships that could be construed as a potential conflict of interest.
