# Supplementary figures and images for: Functional Microbial Responses to Alcohol Abstinence in Patients With Alcohol Use Disorder
Source: Front Physiol. 2020 Apr 24;11:370. doi: 10.3389/fphys.2020.00370 (PMC7193112; doi:10.3389/fphys.2020.00370)

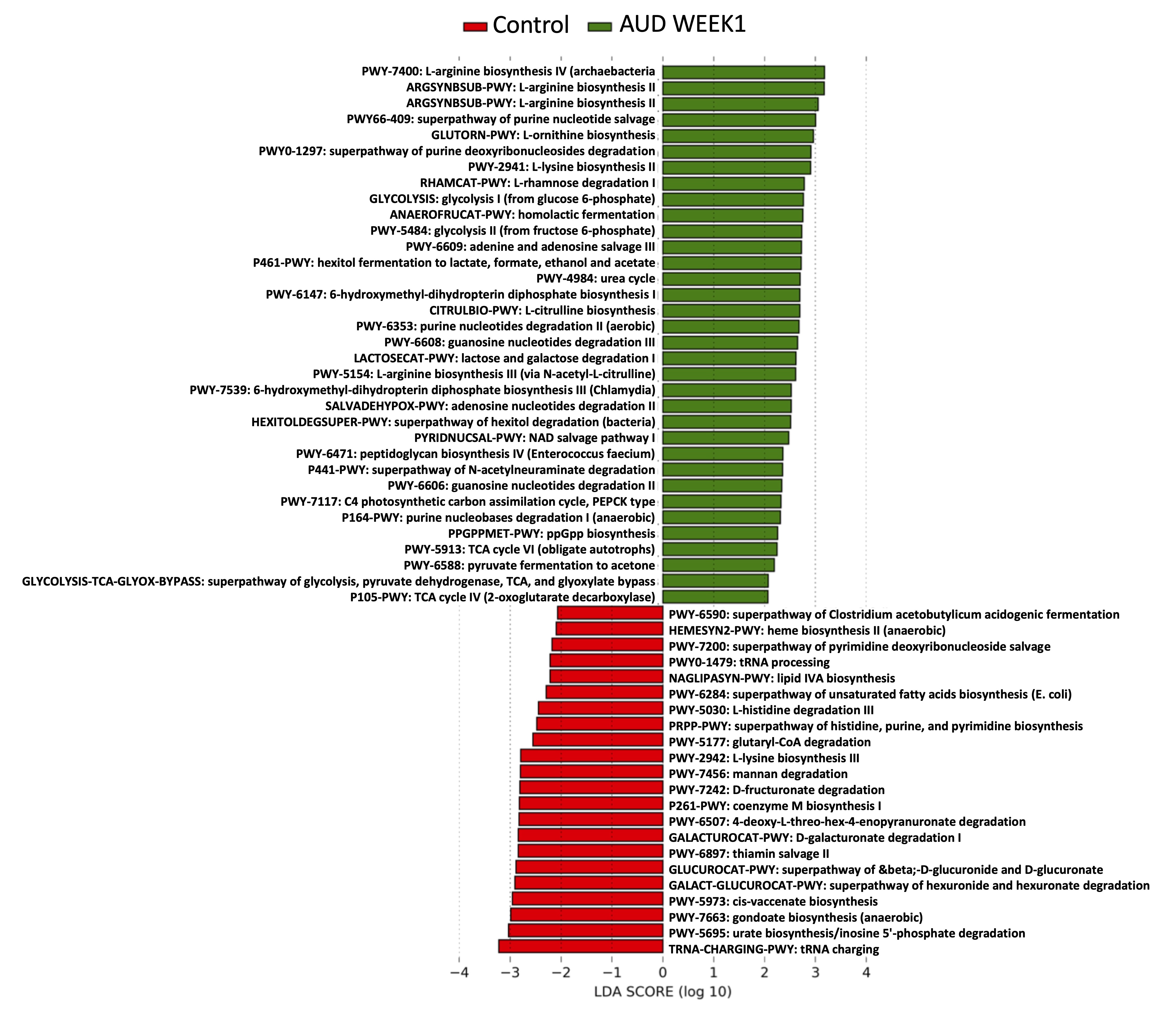

Supplement: Supplementary file 1 [file Image_1.JPEG]

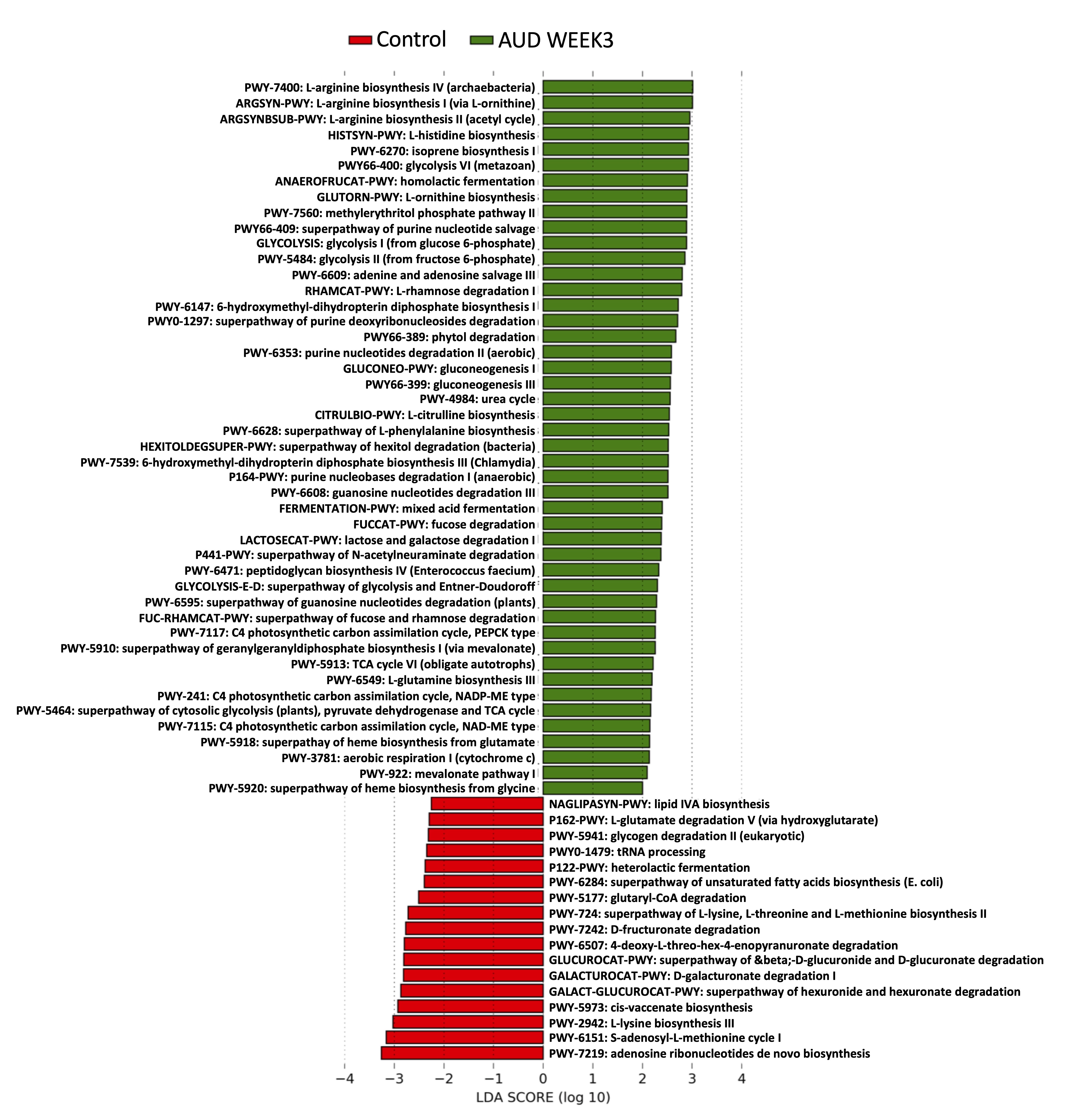

Supplement: Supplementary file 2 [file Image_2.JPEG]

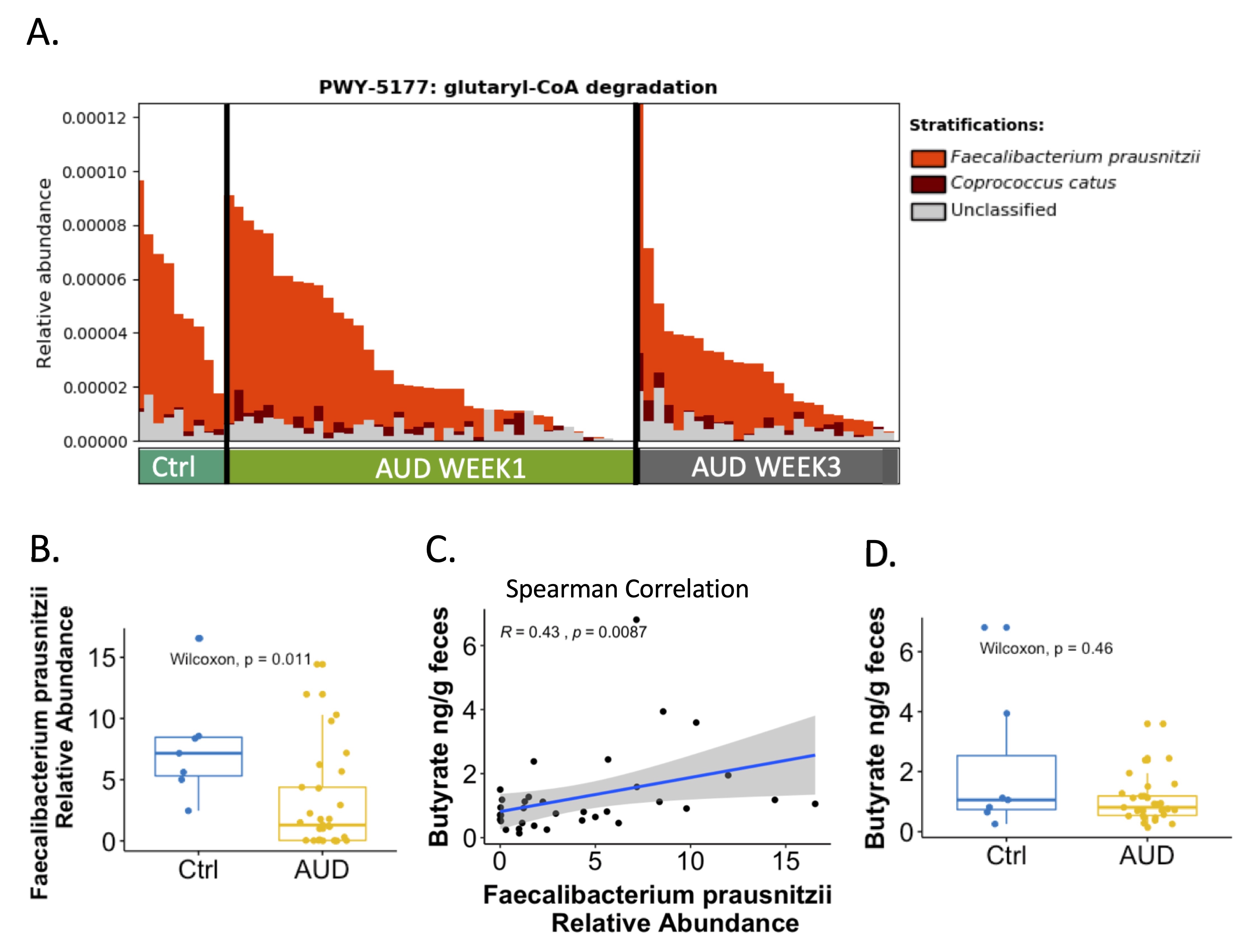

Supplement: Supplementary file 3 [file Image_3.JPEG]
