# Supplementary material for: Functional Microbial Responses to Alcohol Abstinence in Patients With Alcohol Use Disorder
Source: Front Physiol. 2020 Apr 24;11:370. doi: 10.3389/fphys.2020.00370 (PMC7193112; doi:10.3389/fphys.2020.00370)
Supplement: Supplementary file 4 [file Table_1.docx]

**Table S1.** Patient characteristics for the metagenomic analysis

|  | Non-alcoholic Controls | Alcohol Use Disorder  Total | Alcohol Use Disorder  CAP low | Alcohol Use Disorder  CAP high | *p*-value |
| --- | --- | --- | --- | --- | --- |
| Clinical parameter |  |  |  |  |  |
| Total n | 8 | 30 | 17 | 13 |  |
| Age, years, n=38 | 52 (37-71) | 42 (27-59) | 44 (27-59) | 38 (27-53) | 0.12 |
| Body Mass Index (BMI), kg/m², n=38 | 24 (19-29) | 23 (18-31) | 24 (19-31) | 22 (18-25) | **0.02** |
| Gender (male), n(%), n=38 | 6 (75%) | 23 (77%) | 14 (82%) | 9(69%) | 0.71 |
| Laboratory parameter |  |  |  |  |  |
| Albumin (g/dl), n=25 |  | 4.7 (3.9-5.2) | 4.7 (4.2-5.2) | 4.6 (3.9-5.2) | 1 |
| ALT (U/l), n=30 |  | 36 (11-184) | 37 (11-184) | 28 (17-143) | 0.49 |
| AST (U/l) , n=30 |  | 41 (15-283) | 52 (15-155) | 36 (20-283) | 0.79 |
| Total bilirubin (mg/dl), n=27 |  | 0.4 (0.2-1.1) | 0.5 (0.2-1.1) | 0.4 (0.2-0.7) | 0.19 |
| GGT (U/l), n=27 |  | 41 (4-952) | 42 (4-952) | 38(14-558) | 0.86 |
| Platelet counts (x10 9/l), n=26 |  | 223 (21-434) | 250 (21-434) | 222 (80-362) | 0.61 |
| Creatinine (mg/dl), n=27 |  | 0.8 (0.5-1.2) | 0.8 (0.5-0.9) | 0.7 (0.5-1.2) | 0.09 |
| INR, n=27 |  | 0.9 (0.8-1.1) | 0.9 (0.8-1.0) | 0.9 (0.9-1.0) | 0.64 |
| ASCA, n=38 | 9.1 (5.9-20.5) | 12.8 (4.5-37.0) | 12.2 (5.1-37.0) | 13.6 (4.5-24.2) | 0.48 |
| Zonulin, n=38 | 8.4 (4.7-14.9) | 5.2 (1.7-9.8) | 5.3 (2.4-8.8) | 5.0 (1.7-9.8) | 0.06 |
| LBP, n=38 | 9.3 (1.0-12.0) | 7.6 (1.4-25.3) | 7.5 (1.4-25.3) | 7.7 (1.8-18.5) | 0.83 |
| Transient elastography (kPa), n=30 | 5.3 (3.2-6.8) | 5.6 (3.1-7) | 5.3 (3.2-6.8) | 5.9 (3.1-7) | 0.66 |

**Note**: Values are presented as median and range in brackets. The number of patients for which the respective data was available is indicated in the first column. Wilcoxon test was used for comparison between two groups and Kruskal-Wallis test was used for comparison between three groups with Tukey´s post-hoc test for multiple comparisons for continuous variables and Chi-squared tests for categorical variables. Bold font indicates significance (*p*-value <0.05). BMI: CAP high v.s Ctrl 0.09; CAP low v.s. Ctrl 0.99; CAP low v.s. CAP high 0.02. ALT, alanine aminotransferase; AST, aspartate aminotransferase; BMI, body mass index; GGT, gamma-glutamyl-transferase; INR, international normalized ratio; ASCA: Acyl-CoA synthetase short-chain family member 2; LBP: Lipopolysaccharide binding protein.

**Supplemental figure legends**

**Figure S1**. LEfSe analysis of microbial pathways compared between control subjects and alcohol use disorder patients at WEEK1. Red: Increased microbial pathways in control subjects. Green: microbial pathways enriched in alcohol use disorder patients at WEEK1.

**Figure S2**. LEfSe analysis of microbial pathways compared between control subjects and alcohol use disorder patients at WEEK3. Red: Increased microbial pathways in control subjects. Green: microbial pathways enriched in alcohol use disorder patients at WEEK3.

**Figure S3**. *Faecalibacterium prausnitzii* and butyrate. (A) *F. prausnitzii* was the major contributor to glutary-CoA degradation pathway. (B). Relative abundance of *F. prausnitzii* was dereased in alcohol use disorder patients compared with control subjects. (C) Positive spearman correlation was found between the relative abundance of *F. prausnitzii* and fecal butyrate. (D) Butyrate in the fecal samples was not significantly altered between control subjects and alcohol use disorder patients.
